# Pathology characteristics of ocular von Hippel-Lindau disease with neovascularization of the iris and cornea: a case report

**DOI:** 10.1186/s13256-015-0539-2

**Published:** 2015-03-25

**Authors:** Shida Chen, Emily Y Chew, Chi-Chao Chan

**Affiliations:** 1 Immunopathology Section, Laboratory of Immunology, National Eye Institute, National Institutes of Health, Bethesda, MD USA; 2 Zhongshan Ophthalmic Center, Sun Yat-sen University, Guangzhou, China; 3 Division of Epidemiology and Clinical Research, National Eye Institute, National Institutes of Health, Bethesda, MD USA

**Keywords:** Cornea, Iris, Neovascularization, Retinal hemangioblastoma, von Hippel-Lindau disease

## Abstract

**Introduction:**

Retinal hemangioblastoma is one of the most common tumors in von Hippel-Lindau disease. In addition to the classical pathological characteristics of von Hippel-Lindau disease, we report, for what we believe to be the first time, a severe and rare ocular complication characterized by neovascularization in the cornea and iris.

**Case presentation:**

A 41-year-old white man with a long history of retinal hemangioblastoma presented with neovascularization of his iris and cornea as well as corneal perforation. His right eye was blind and painful, leading to a decision of enucleation. On microscopy, the enucleated eye showed neovascularization of the cornea and iris. The cornea was perforated with an expulsive hemorrhage and extruding intraocular contents, including the retina. A large retinal hemangioblastoma was located at the posterior pole adjacent to the optic nerve head. The tumor was mainly composed of large cells with foamy cytoplasm. Bone formation was also present.

**Conclusion:**

Our pathology findings were consistent with previously described features of retinal hemangioblastoma. The present case is unusual because of the co-existing neovascularization in the iris and cornea, which may have led to corneal perforation and vision loss.

## Introduction

Von Hippel-Lindau disease (VHL) is an autosomal dominant systemic syndrome that results from a mutation in the *VHL* gene on chromosome 3 (3p25-26) [1]. Disruption of VHL protein function leads to an accumulation of hypoxia-inducible transcription factor 1α (HIF-1α), which induces overproduction of its target genes, including vascular endothelial growth factor (*VEGF*), platelet-derived growth factor - beta (*PDGFB*), and transforming growth factor alpha (*TGFA*). The growth factors are also shown to contribute to the formation of tumors [1,2]. Retinal hemangioblastoma is seen in more than 60% of patients with VHL disease [3]. Approximately half of patients with retinal hemangioblastoma have bilateral involvement. The prominent ocular complications of retinal hemangioblastoma are retinal exudate and tractional retinal detachment [4]. On pathology, retinal hemangioblastoma appears as a network of thin vascular capillary-like channels lined by endothelial cells and pericytes. These vascular channels are separated by foamy VHL-associated tumor cells, also known as stromal cells [5,6]. Complications outside the retina are uncommon in VHL disease.

We report, for what we believe to be the first time, the pathological characteristics of a case of retinal hemangioblastoma with neovascularization involving the iris and cornea. The study was approved by the National Eye Institute Institutional Review Board for human subjects, and our patient signed an informed consent.

## Case presentation

**Figure 1:**
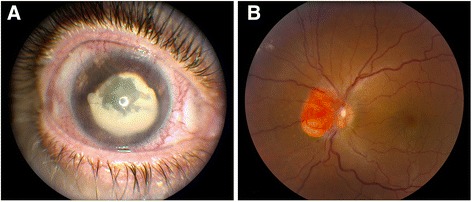
**Clinical photographs of the eye from the patient with von Hippel-Lindau disease. (A)** The right eye shows neovascularization of the cornea and iris (rubeosis iridis) and matured cataract. **(B)** The left eye shows a hemangioblastoma involving the optic nerve.

A 41-year-old white man was diagnosed with VHL with multiple retinal hemangioblastomas in 1987 at the age of 17 years. He received thermal laser and cryotherapy treatment for a retinal hemangioblastoma in his right eye in 1992. The tumor progressed and upon examination in June 2007 he had no light perception in his right eye, with a completely obscured fundus. On examination in April 2011, there was still no light perception in his right eye; his intraocular pressure was 52mmHg; and band keratopathy, rubeosis iridis, and dense cataracts were present (Figure 1A). On examination in July 2012, his right eye was blind and painful with an intraocular pressure of 48mmHg and a corneal ulcer. The vision in his left eye was 20/20, and the fundus showed evidence of a retinal hemangioblastoma involving the optic nerve and the presence of retinal exudates (Figure 1B). He underwent enucleation of the blind and painful right eye in November 2013.

**Figure 2:**
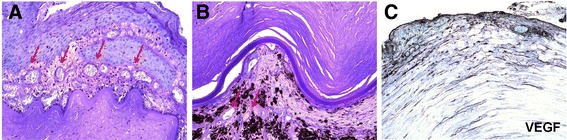
**Photomicrographs of neovascularization in the cornea and iris. (A)** The corneal epithelium shows epidermalization. Many small vascular lumina (arrows) are located in the corneal subepithelial region. **(B)** Small vascular lumina (arrows) are present superficially and in the surface of the iris. **(C)** The anterior cornea shows high expression of vascular endothelial growth factor (VEGF). **(A** and **B**, hematoxylin and eosin, original magnification, ×200; **C**, avidin-biotin complex immunohistochemistry, original magnification, ×200).

The enucleated eye was sent to the National Eye Institute for pathological examination. Routine histopathology and immunohistochemistry were performed on the enucleated right globe. Macroscopically, the cornea was perforated by an expulsive hemorrhage. The anterior chamber was completely occluded by a pupillary membrane admixed with intraocular contents and extensive hemorrhaging. The vitreous cavity was filled with hemorrhage, and the retina was poorly identified. There was bone tissue admixed with hemorrhages in the posterior pole. The optic nerve also contained hemorrhage. On microscopy, the cornea was perforated centrally, where the hemorrhage was mixed with the exposed intraocular contents including the uvea and retina. Most of the remaining corneal epithelium showed changes in epidermalization, and there was extensive neovascularization with small hemorrhages at the anterior corneal surface (Figure 2A). Consequently, immunostaining for VEGF was positive at the corneal surface (Figure 2C). The atrophic iris was disorganized and adhered to the Descemet’s membrane. Additionally, the surface of the iris showed neovascularization (Figure 2B). The retina was totally detached, disorganized, and showed marked gliosis.

**Figure 3:**
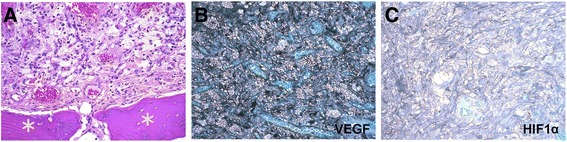
**Photomicrographs of retinal hemangioblastoma. (A)** The retinal hemangioblastoma was composed of classical foamy (tumor) cells admixed with small capillaries. Osseous tissues (asterisks) are adjacent to the retinal hemangioblastoma. **(B)** The hemangioblastoma shows high expression of vascular endothelial growth factor (VEGF). **(C)** Hypoxia-inducible transcription factor 1α (HIF-1α) is expressed in the retinal hemangioblastoma. **(A**, hematoxylin and eosin, original magnification, ×200; **B** and **C**, avidin-biotin complex immunohistochemistry, original magnification, ×200).

A large retinal hemangioblastoma was noted at the optic nerve head (Figure 3A). The tumor was mainly composed of large cells with foamy cytoplasm (Figure 3A). Within the retinal hemangioblastoma, there were focal areas of cystic degeneration, gliosis, and hemorrhage. There was osseous transformation beneath the hemangioblastoma, mixed with gliofibrous tissues in some areas (Figure 3A). Expression of VEGF and HIF-1α was strongly positive within the hemangioblastoma and in the optic nerve (Figure 3B,C). Large hemorrhages beneath the choroid were observed.

## Discussion

Ocular complications of retinal hemangioblastoma are predominantly exudative (25%) or involve tractional retinal detachment (9%) [7]. Retinal or vitreous hemorrhages are rare and only occur in fewer than 3% of cases; anterior segment complications such as cataract and neovascular glaucoma are also rare but have been found to occur during the end-stage of the disease [4,7]. Though ocular VHL disease can sometimes manifest as retinal neovascularization characterized by fine, superficial vascular proliferation [8], neovascularization in non-retinal tissues is rare. It has been found that only 2% of eyes with retinal hemangioblastomas also show neovascularization in the iris [4]. To the best of our knowledge, no cases of severe corneal neovascularization and perforation associated with retinal hemangioblastoma have been reported. Although retinal capillary hemangioblastomas are slow growing and benign in nature, they can sometimes result in vision loss and disruption of the structural integrity of the globe through exudative and tractional effects on the retina [4]. This type of severe visual impairment is more likely to be associated with increased age, the presence of juxtapapillary lesions, and the increased number and extent of peripheral lesions [3]. In our case, his right eye developed total retinal detachment, hemorrhage in the vitreous and subretinal space, neovascularization of the iris and cornea, and corneal perforation; the combination of these symptoms led to the painful blind eye.

Typically, retinal hemangioblastomas comprise capillary-like vascular channels surrounded by large vacuolated stromal cells [9,10]. In our case, we found similar pathological features in the retinal hemangioblastoma as well as osseous bone formation around the tumor, correlating with the prolonged history of the disease.

High levels of VEGF are believed to contribute to hemangioblastoma formation; in animal models where VEGF is overexpressed or where VHL is inactive, visceral lesions resembling hemangioblastomas form [11,12]. High levels of VEGF have also been reported in the ocular fluid and tumors of patients with VHL [6,13]. We detected high expression of VEGF at the site of corneal neovascularization and within the retinal hemangioblastoma of our patient. Further, HIF-1α expression was increased within the retinal hemangioblastoma, which is consistent with previous findings that there are many HIF transcripts in retinal hemangioblastomas [14]. This information suggests there may be potential therapeutic implications of targeting HIF-1α in VHL disease [15].

## Conclusion

Co-existing ocular complications of the retinal hemangioblastoma occur relatively infrequently. We present a case with these clinicopathological features, including neovascularization of the iris and cornea.

## Consent

Written informed consent was obtained from the patient for publication of this case report and the accompanying images. A copy of the written consent is available for review by the Editor-in-Chief of this journal.

## Abbreviation

HIF-1α: hypoxia-inducible transcription factor α
VEGF: vascular endothelial growth factor
VHL: von Hippel-Lindau disease
